# Anorectal Melanoma: An Uncommon Cause of Lower Gastrointestinal Bleeding

**DOI:** 10.7759/cureus.16821

**Published:** 2021-08-02

**Authors:** Nada M Alsharif, Haya Omeish, Mohammad Abdulelah, Mohammed A Abu-Rumaileh, Husam Bader

**Affiliations:** 1 Department of Internal Medicine, King Hussein Cancer Center, Amman, JOR; 2 Department of Internal Medicine, Royal Jordanian Medical Centre, Amman, JOR; 3 Department of Internal Medicine, The University of Jordan, Amman, JOR; 4 Department of Internal Medicine, Presbyterian Medical Center, Albuquerque, USA

**Keywords:** elderly, melanoma, anorectal melanoma, anorectal malignancy, irritable bowel disease, lower gastrointestinal bleeding

## Abstract

Anorectal melanoma (ARM) is a rare, aggressive disease. Given that it presents with local symptoms that resemble other common benign anorectal conditions, ARM is often low on the differential diagnosis. Delayed diagnosis and nonconsensus of treatment options lead to poor prognosis. Here, we report the case of an 85-year-old woman with a history of Irritable bowel syndrome who presented with altered bowel habits and bleeding per rectum. CT revealed a rectal mass with metastatic lesions to the bone, liver, and lungs. Immunohistochemical staining was positive for Human Melanoma Black-45, melanoma antigen recognized by T cells, and SRY-related HMG-box 10. A final diagnosis of ARM was made.

## Introduction

Lower GI bleeding is a common presentation in the emergency department and clinic settings. Common etiologies are often grouped into benign conditions (such as hemorrhoids, diverticulosis, infections, and arteriovenous malformations) or malignant causes (such as colorectal carcinoma). However, for a broad differential diagnosis, other less common etiologies may be considered including uncommon malignancies (melanomas, lymphomas), infiltrative pathologies (amyloidosis), and vascular abnormalities (Dieulafoy’s lesions). Anorectal melanoma (ARM) is a rare disorder, with a 0.5-4% incidence rate of all malignant tumors in the region [1,2]. Mucosal melanoma represents the third most common site of malignant melanomas; most commonly, melanoma is of the cutaneous subtype with more than 90% of cases, followed by ocular melanoma [3]. ARM is more common in women, and its incidence is currently on the rise in the elderly [4,5], with more than 65% of cases diagnosed in patients over 60 years of age [6]. Due to its rarity and similarity in presentation to other more benign and common conditions such as hemorrhoids, diagnosis of ARM is usually delayed leading to poor outcomes. Overall, 60% of patients have lymph node involvement on initial presentation and 20% have distant metastatic lesions [7], leading to a poor prognosis with almost no patients surviving stage 3 disease for more than five years [8,9].

## Case presentation

**Figure 1:**
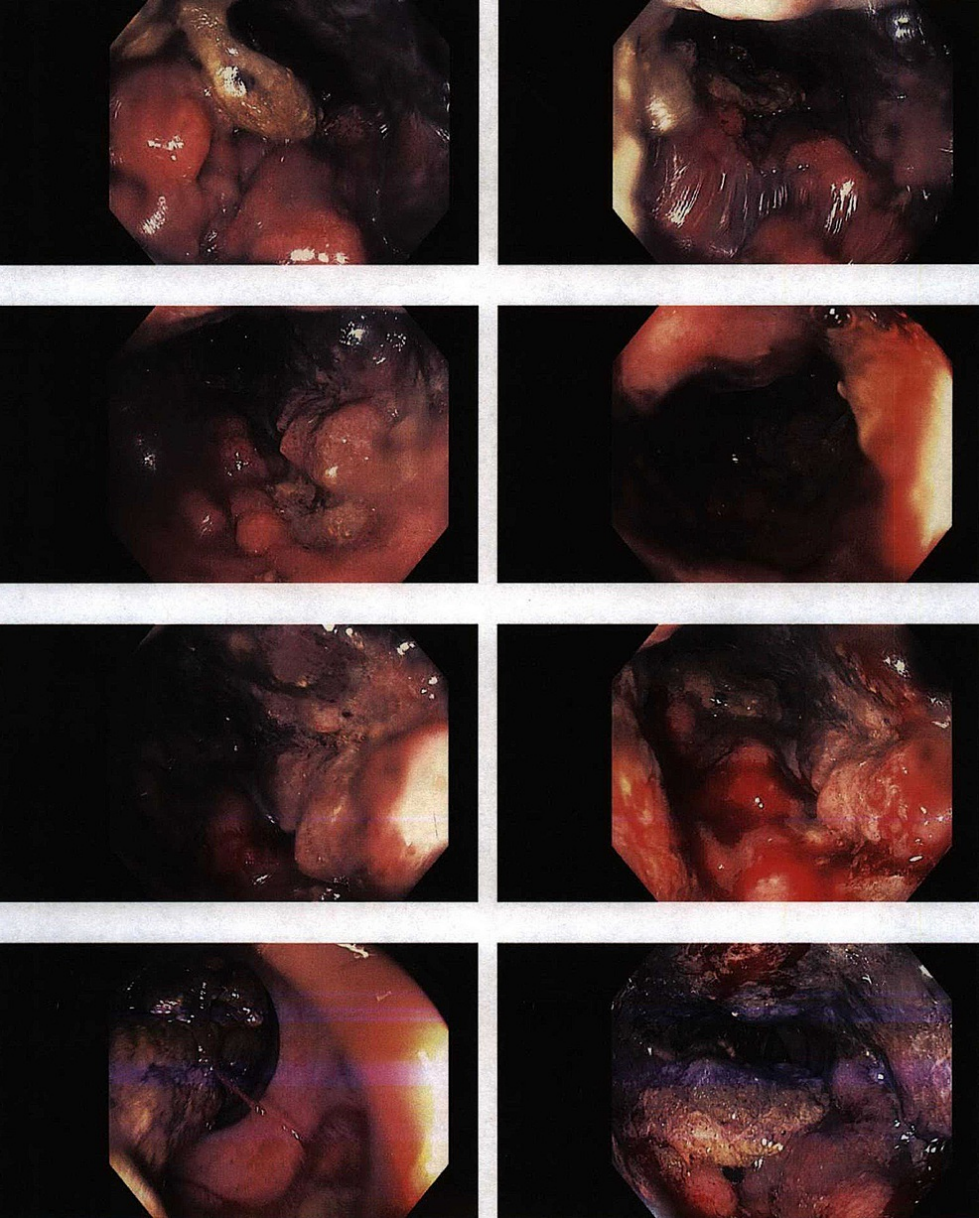
Colonoscopy showing a mass with erythematous, congested, and friable mucosa that appeared fungating and partially obstructing the lumen (white arrows).

An 85-year-old woman presented to the outpatient clinic with bright red blood per rectum that occurred with and without bowel movements. She reported having intermittent rectal bleeding for a month that became persistent. In addition, she complained of diarrhea alternating with constipation for several months with varying frequency, along with a feeling of pressure in the pelvis with defecation. Physical examination was unremarkable. A computed tomography (CT) scan of the chest/abdomen/pelvis revealed a distal rectal mass, with severe circumferential mucosal thickening extending over 8 cm and multiple enlarged lymph nodes in a pattern highly suggestive of rectal carcinoma. Metastatic lesions in the liver, lungs, and bone were detected. Colonoscopy showed a mass with erythematous, congested, and friable mucosa that appeared fungating (Figure 1). It partially obstructed the lumen and extended from the dentate line to 20 cm. Biopsy of the rectal mass was performed which showed large, poorly cohesive cells with amphophilic cytoplasm and variable intracytoplasmic melanin pigment. These cells were found positive for Human Melanoma Black-45 (HMB-45), melanoma antigen recognized by T cells (MART-1), and SRY-related HMG-box 10 (SOX-10) on immunohistochemistry. A diagnosis of ARM was made. Tumor serum markers for BRAF and Kit were both negative. The patient was referred to an oncologist for treatment and palliative radiation was planned.

## Discussion

Melanomas originate from melanocytic cells which can be found throughout the body, most commonly in the skin. In the rectum, these cells are located around the dentate line, and when malignant, they spread submucosally. Diagnosis of ARM can be challenging as it can resemble other adenocarcinomatous malignancies on histopathology [10]. Moreover, up to 20% of lesions can be amelanocytic [11,12]. Immunohistochemistry staining for melanoma markers (e.g., HMB-45, MART-1, and S100) has proven crucial in making the right diagnosis. More cases of ARM, which is a rare, aggressive disease, are being reported in the literature [13-15]. It presents with rectal bleeding, tenesmus, altered bowel movements, and an anorectal mass, all of which may overlap with more common benign conditions such as hemorrhoids, rectal polyps, and irritable bowel syndrome (IBS). Thus, more cases are diagnosed and treated late due to this resemblance. Initial diagnoses may be incorrect in 80% of ARM cases [10,16]. Our patient initially complained of altered bowel movements for several months, which was attributed to her IBS and managed accordingly. It was not until she developed bleeding per rectum requiring further workup that the final diagnosis was made. The highly vascular and lymphatic supply of the anus leads to a more aggressive disease. Most patients already have metastatic disease at the time of presentation, as seen in our patient who presented with multiple metastases to the bone, lung, and liver.

Treatment of ARM is still debatable because of the rarity of the condition and the few studies comparing different modalities of treatment. Surgery, chemotherapy, and radiotherapy have been implicated. While surgery remains the mainstay of treatment, which varies from wide local excision to abdominoperineal resection, the therapeutic benefit of these procedures remains questionable [17,18]. Unfortunately, due to the late widespread disease at the time of presentation, our patient’s options were limited to palliation to limit the progression of disease and control symptoms. The patient opted against any treatment with curative intent.

## Conclusions

The high vascularity of the anus contributes to the aggressive nature of ARM, therefore, most patients present at an advanced stage. Presentation is often nonspecific and varies from common symptoms such as altered bowel movement to red flags such as anorectal mass and rectal bleeding, which makes the diagnosis challenging. We encourage physicians to widen their differential diagnoses and have a lower threshold for malignant etiologies in high-risk individuals, particularly in individuals with previously stable or mild IBS who present with new GI symptoms. There is no consensus regarding the treatment plan for ARM. Surgery, chemotherapy, and radiotherapy are treatment options. Further studies are recommended to decrease both the morbidity and mortality rates and achieve better care for patients.
